# A novel approach to remove the batch effect of single-cell data

**DOI:** 10.1038/s41421-019-0114-x

**Published:** 2019-09-24

**Authors:** Feng Zhang, Yu Wu, Weidong Tian

**Affiliations:** 1 State Key Laboratory of Genetic Engineering and Collaborative Innovation Center for Genetics and Development, School of Life Sciences, Fudan University, 200436 Shanghai, P.R. China; 2 Department of Biostatistics and Computational Biology, School of Life Sciences, Fudan University, 200436 Shanghai, P.R. China; 3 Department of Pediatrics, Brain Tumor Center, Division of Experimental Hematology and Cancer Biology, Cincinnati Children’s Hospital Medical Center, Cincinnati, OH 45229 USA; 4 Children’s Hospital of Fudan University, 201102 Shanghai, China

**Keywords:** Bioinformatics, Transcription

Dear Editor,

Analyzing single-cell RNA sequencing (scRNA-seq) data from different batches is a challenging task^1^. The commonly used batch-effect removal methods, e.g. Combat^2,3^ were initially developed for microarray or bulk RNA-seq data, and may not be appropriate for single-cell analysis in some situations^4^. Recently, several batch-effect removal tools specific for single-cell data have been developed. One of them is called canonical correlation analysis (CCA) subspace alignment (implemented in Seurat)^4^, which conducts CCA and uses dynamic time warping to align the subspaces of different batches. However, CCA may lose the subspaces with the largest possible variance (can be identified by PCA), leading to wrong alignment result when the cell types of different batches are extremely imbalanced. To remove batch-effect from the PCA subspaces based on the correct cell alignment, a method called fastMNN^5^ detects mutual nearest neighbors (MNN) of cells in different batches, and then uses the MNN to correct the values in each PCA subspace. Although fastMNN was shown to have a good performance, in practice it has long running time, and also lacks the explainability because of the correction of values in PCA subspace. A graph-based method named batch balanced KNN (BBKNN)^6^ reduces batch-effect by creating connections between analogous cells in different batches. However, BBKNN only generates the final vectors (UMAP)^7^, making it impossible to track the adjustment. In this study, we present a novel method called batch effect remover (BEER) for combining scRNA-seq data from different batches. The originality of BEER is that it uses the correlation of mutual nearest (MN) cell pairs identified from different batches to identify PCA sub-spaces with poor correlation (i.e., latent high batch-effect), and then removes these subspaces from further analysis. Because BEER does not change any values in PCA subspaces, the results produced by BEER are trackable and easily explainable. By using a cell-type imbalanced benchmark, we show that BEER has a clear advantage over four representative batch-effect removal tools: Combat, Seurat (CCA alignment), fastMNN, and BBKNN.

BEER has been implemented in R. The inputs of BEER are two expression matrices (UMI or other un-scaled expression format) coming from two different batches. The row and column names of the two expression matrices are gene and cell names, respectively. The workflow of BEER includes two main parts (Fig. 1a). In the first part, for each expression matrix, BEER preprocesses the data and conducts t-distributed stochastic neighbor embedding (tSNE)^8^ to transfer the data into one-dimension values. tSNE is used to do one-dimension reduction because of its robustness and well-recognized performance in the field of scRNA-seq analysis^9^. BEER groups cells (default number of cells in each group is 10) based on the order of the one-dimension values, and then aggregate the expression profiles of each cell in a group to obtain the representative expression profile for that group. Next, BEER calculates a Kendall’s tau to evaluate the distance of each pair of cell group from two batches, and identifies all MN pairs of cell groups in between the two batches. In the second part, BEER directly combines two expression matrices, normalizes the data, and conduct PCA to produce a number (default is 50) of subspaces. Because two cell groups in a MN-paired cell groups represent the most similar groups in those two batches, they should have similar values in each PCA subspace if there is no batch effect. Thus, by calculating the correlation between MN-paired cell groups in each subspace, BEER identifies those with poor correlation and considers them to have latent high batch-effect. Finally, BEER simply removes those PCA subspaces with latent batch effect, and no values in the other subspaces are changed (details are provided in Supplementary information). Note that it is likely that a removed PCA subspace may also have biological variances. A workflow has been provided to help users determine whether a PC removed by BEER has biological meaning (see Supplementary information for the workflow); then, other methods, such as ComBat, may be used to modify this PC.

**Fig. 1:**
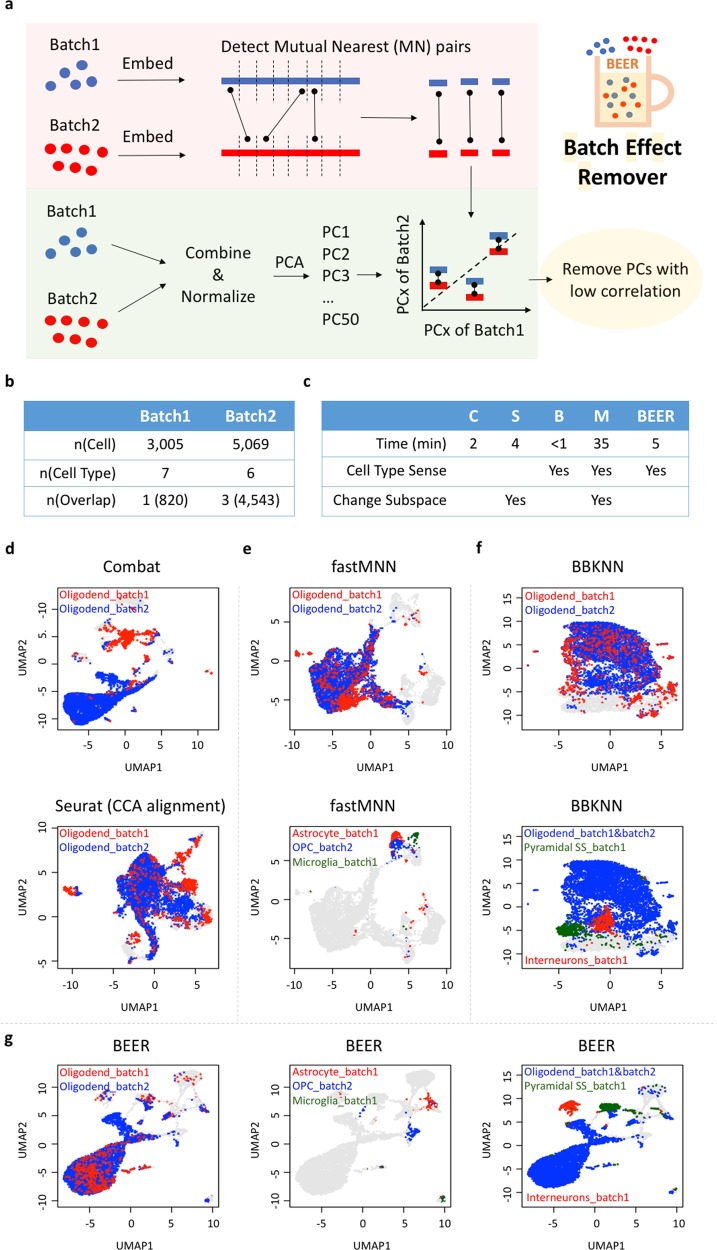
Workflow and benchmark study of BEER. **a** Shows the workflow of BEER. In the “Embed” step, we use tSNE to transfer single-cell expression matrix into one-dimension values. When detecting mutual nearest (MN) pairs, we use Kendall’s tau (“cor.fk” function of “pcaPP” package in R) to evaluate the distance (higher Kendall’s tau means shorter distance). We use “cor.test(method = ’kendall’)” in R to test the correlation between MN-paired cell groups. Details are provided in Supplementary information. **b** Shows the basic information of the benchmark data sets. “Batch1” is derived from a cortex study^10^, while “Batch2” is derived from an oligodendrocyte study^11^. The third row shows the number of cell types (or cells in parenthesis) in “Oligodendrocytes”. Details about those two batches are in Supplementary information. **c** Shows the summary of the methods being compared in this study. “C”, “B”, “S”, and “M” stand for “Combat”, “BBKNN”, “Seurat (CCA alignment)”, and “fastMNN”, respectively. “Cell Type Sense” means that the method can sense same-type cells across different batches. “Change Subspace” means that the method changes the values of PCA (or CCA) subspace. Details about the competing methods are in Supplementary information. **d–g** The UMAP figures show the output of each method. For figures with “Oligodend_batch1” and “Oligodend_batch2”, the red and blue points are oligodendrocytes in batch1 and batch2, respectively. Figures with three labels show the location of three different cell types that should be separated due to their biological difference in UMAP. UMAP figures with all cell-type labels in high resolution are shown in Supplementary information

We apply BEER and other four representative batch-effect removal methods (Combat, BBKNN, Seurat CCA alignment, and fastMNN) to a stringent cell-type imbalanced benchmark. In this benchmark, there are two batches: one is from a mouse cortex study^10^, and another is from a mouse oligodendrocyte study^11^. Except the cell type named “Oligodendrocytes”, the other cell types of those two batches are completely different (Fig. 1b and Supplementary information). The total number of cells in this benchmark is 8074. The running time of almost all methods is about 1–5 min, while fastMNN uses 35 min (Fig. 1c). We apply UMAP to visualize the output of each method. As can be seen in Fig. 1d, Combat and Seurat (CCA alignment) fail to mix oligodendrocyte cells from the two batches. Although oligodendrocyte cells from the two batches are mixed by fastMNN and BBKNN, these two methods fail to separate biologically different cell types of different batches (Fig. 1e, f): fastMNN mixes Astrocyte_batch1, OPC_batch2, and Microglia_batch1 together (Fig. 1e), while BBKNN mixes Oligodendrocytes_batch1&batch2, Pyramidal SS_batch1, and Interneurons_batch1 together (Fig. 1f). In contrast, BEER not only successfully mixes oligodendrocytes of two different batches together, but also separates the cell types that are not separated by fastMNN and BBKNN into different locations (Fig. 1g), showing a clear advantage over other methods.

We have inspected BEER’s performance to the change of tSNE perplexity values and the change of cell group size (for aggregating expression profiles), and have found that BEER is fairly robust to these changes (Supplementary information). We have also used a quantitative metric-Silhouette coefficient to compare the performance of different methods for removing batch-effects, and have demonstrated that BEER clearly outperforms the other methods (Supplementary information). In addition, for batch-effect removal of more than two batches, we have provided a function named “MBEER” which identifies the batch with the most number of cells as the target batch, and applies BEER iteratively for comparing the other batches with the target batch (for details, see Supplementary information). Alternatively, users can define the target batch, and then apply “MBEER” for batch-effect removal of more than two batches.

In conclusion, BEER has three main features: (a) BEER can mix the same-type cells of different batches without losing the identities of different types of cells in different batches. (b) All steps of BEER are transparent and trackable. (c) BEER is efficient, and the “parallel” package has been implemented in BEER for multi-threads processing. A user guide of BEER is provided in Supplementary information. For convenience, BEER and all scripts of this study are available at https://github.com/jumphone/BEER

## Supplementary information

Supplementary information.

## Data Availability

BEER may be used in R and source code is maintained at https://github.com/jumphone/BEER. All scripts used in this study are available in this repository.

## Supplementary information

**Supplementary Information** accompanies the paper at (10.1038/s41421-019-0114-x).
