# Supplementary material for: A novel approach to remove the batch effect of single-cell data
Source: Cell Discov. 2019 Sep 24;5:46. doi: 10.1038/s41421-019-0114-x (PMC6796914; doi:10.1038/s41421-019-0114-x)
Supplement: Supplementary file 1 — Supplementary information. [file 41421_2019_114_MOESM1_ESM.pdf]

Supplementary information

1. METHODS **2**

2. BENCHMARKS **4**

2.1 MOUSE CORTEX STUDY – ZEISEL ET AL. 4

2.2 MOUSE OLIGODENDROCYTES STUDY – MARQUES ET AL. 4

3. COMPETING TOOLS **5**

4. UMAP FIGURES IN HIGH RESOLUTION **6**

4.1 BEER (PERPLEXITY=30, CELL NUMBER PER GROUP=10) 6

4.2 COMBAT 6

4.3 SEURAT (CCA ALIGNMENT) 7

4.4 FASTMNN 7

4.5 BBKNN 8

4.6 BEER (PERPLEXITY=5, CELL NUMBER PER GROUP=10) 8

4.7 BEER (PERPLEXITY=50, CELL NUMBER PER GROUP=10) 9

4.8 BEER (PERPLEXITY=100, CELL NUMBER PER GROUP=10) 9

4.9 BEER (PERPLEXITY=30, CELL NUMBER PER GROUP=50) 10

5.0 BEER (PERPLEXITY=30, CELL NUMBER PER GROUP=100) 10

5. SILHOUETTE PLOT **11**

6. USER GUIDE OF BEER **11**

6.1 REQUIREMENT 11

6.2 USAGE 11

7. BEER WITH MULTIPLE BATCHES **14**

8. DETECT PCS WITH BOTH BATCH EFFECTS AND BIOLOGICAL VARIANCES **14**

REFERENCES **16**

1. Methods

Here, we provide more details about the workflow of BEER:

*The workflow of BEER includes two main parts (**Fig. 1a**). In the first part, for each ex-pression matrix, BEER preprocesses* **[M1]** *the data and conducts t-distributed Stochastic Neighbor Embedding (tSNE) to transfer the data into one-dimension values. BEER groups cells (default number of cells in each group is 10) based on the order of the one-dimension values, and then aggregate the expression profiles of each cell in a group to obtain the representative expression profile for that group. Next, BEER calculates a Kendall's tau to evaluate the distance of each pair of cell group from two batches and identifies all Mutual Nearest (MN) pairs* **[M2]** *of cell groups in between the two batches. In the second part, BEER directly combines two expression matrices* **[M3]**, *normalizes the data* **[M4]**, *and conduct PCA to produce a number (default is 50) of subspaces. Because two cell groups in a MN-paired cell groups are assumed to be from the same cell type, they are supposed to have similar values in each PCA subspace if there is no batch effect. Thus, by calculating the correlation between MN-paired cell groups in each subspace* **[M5]**, *BEER identifies those with poor correlation and considers them to have latent high batch-effect. Finally, BEER simply removes those PCA subspaces with latent batch effect, and no values in the other subspaces are changed*

Detailed scripts are in: https://github.com/jumphone/BEER/blob/master/BEER.R

M1 (preprocessing): For each inputted expression matrix, we use "Seurat" package in R to conduct normalization. At first, we use the function named "NormalizeData" to normalize ("LogNormalize', scale.factor=10000) the data. Then, we use the function called "ScaleDate" to standardize [vars.to.regress = c("nUMI")] the data. Finally, we use "RunPCA" to calculate a number (default is 50) of PCA subspaces and use those PCA subspaces to conduct the following t-distributed Stochastic Neighbor Embedding (tSNE).

M2 (Mutual Nearest): For the definition of Mutual Nearest, please refer to the **Figure 1** of Haghverdi et al.'s paper (Batch effects in single-cell RNA-sequencing data are corrected by matching mutual nearest neighbors, Nature Biotechnology, 2018) (Haghverdi, et al., 2018).

M3 (Combine Data): We simply combine two expression matrices. Those overlapped genes are used to generate the combined matrix.

M4 (Normalization of Combined Data): We use the function named "FindVariableGenes" in "Seurat" to identify variable genes (default parameters). For other steps, please refer to **M1**.

M5 (Correlation Test): For each subspace, we generate two subspace-value lists. The first and the second list are prepared for Batch1 and Batch2, respectively. For each MN-paired group, we use "quantile" function in R to get five values of each batch, and then append those quantile

values of Batch1 and Batch2 to the end of the first and the second subspace-value list, respectively. After going through all MN-paired groups, we use “cor.test(method=’kendall’)” in R to test the correlation between those two subspace-value lists.

2. Benchmarks

2.1 Mouse Cortex Study – Zeisel et al.

This benchmark has 3,005 mouse brain cells and 3,752 sequenced genes per cell (Zeisel, et al., 2015). We download the expression matrix (count) from:

https://storage.googleapis.com/linnarsson-lab-www-blobs/blobs/cortex/expression_mRNA_17-Aug-2014.txt

Cell Types:

**Type**	**n(Cell)**
astrocytes_ependymal	224
Oligodendrocytes	820
microglia	98
endothelial-mural	235
interneurons	290
pyramidal CA1	939
pyramidal SS	399

2.2 Mouse Oligodendrocytes Study – Marques et al.

This benchmark has 5,069 mouse oligodendro lineage cells and 2,570 sequenced genes per cell (Marques, et al., 2016). In this study, "Newly-formed Oligodendrocytes", "Myelin-forming Oligodendrocytes", and "Mature Oligodendrocytes" are all defined as "Oliodendrocytes". We download the expression matrix (count) from:

https://www.ncbi.nlm.nih.gov/geo/query/acc.cgi?acc=GSE75330

Cell Types:

**Type**	**n(Cell)**
PPR	76
OPC	310
COP	140
Newly-formed Oligodendrocytes	512

Myelin-forming Oligodendrocytes	1283
Mature Oligodendrocytes	2748

3. Competing Tools

In this study, we compared BEER with four other methods: Combat (Johnson, et al., 2007), BBKNN (Park, et al., 2018), Seurat (CCA alignment) (Butler, et al., 2018), and fastMNN (Haghverdi, et al., 2018). Combat is built in an R package named "sva" (https://bioconductor.org/packages/release/bioc/html/sva.html). BBKNN is downloaded from https://github.com/Teichlab/bbknn. Seurat (CCA alignment) is downloaded from https://satijalab.org/seurat/install.html. FastMNN is a function in an R package named "scran" (http://bioconductor.org/packages/release/bioc/html/scran.html). All scripts of running those four methods are shown in https://github.com/jumphone/BEER/tree/master/Benchmark.

4. UMAP Figures in High Resolution

4.1 BEER (perplexity=30, cell number per group=10)

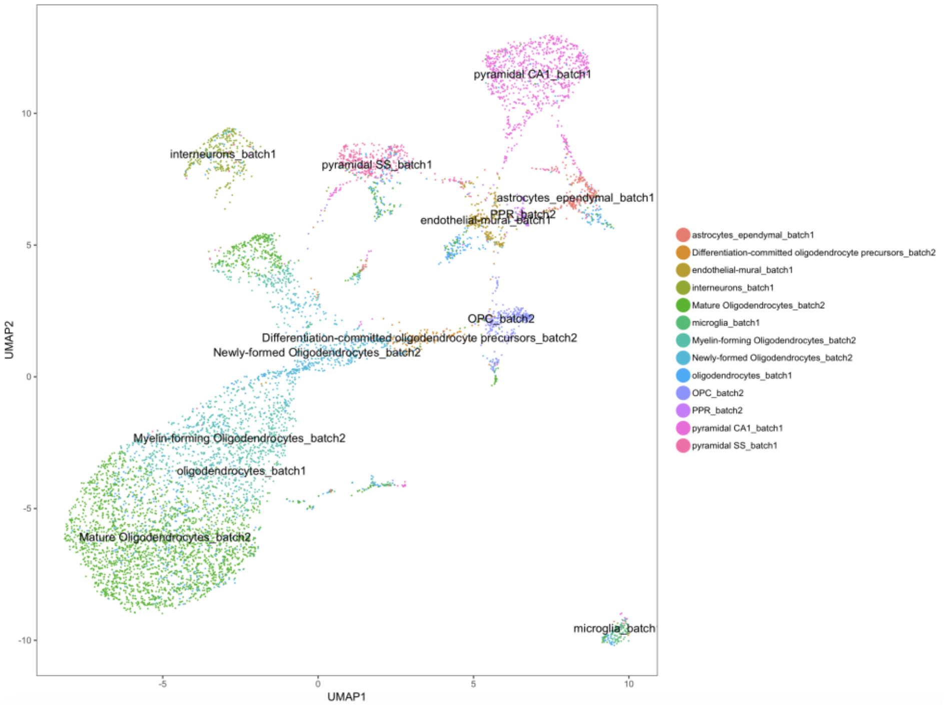

4.2 Combat

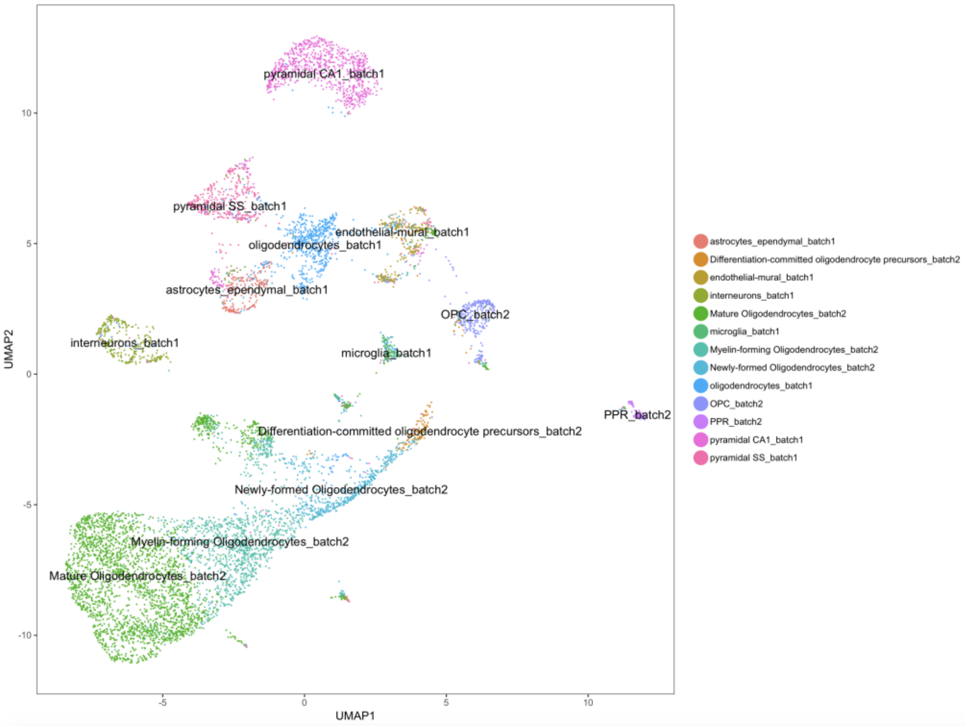

4.3 Seurat (CCA alignment)

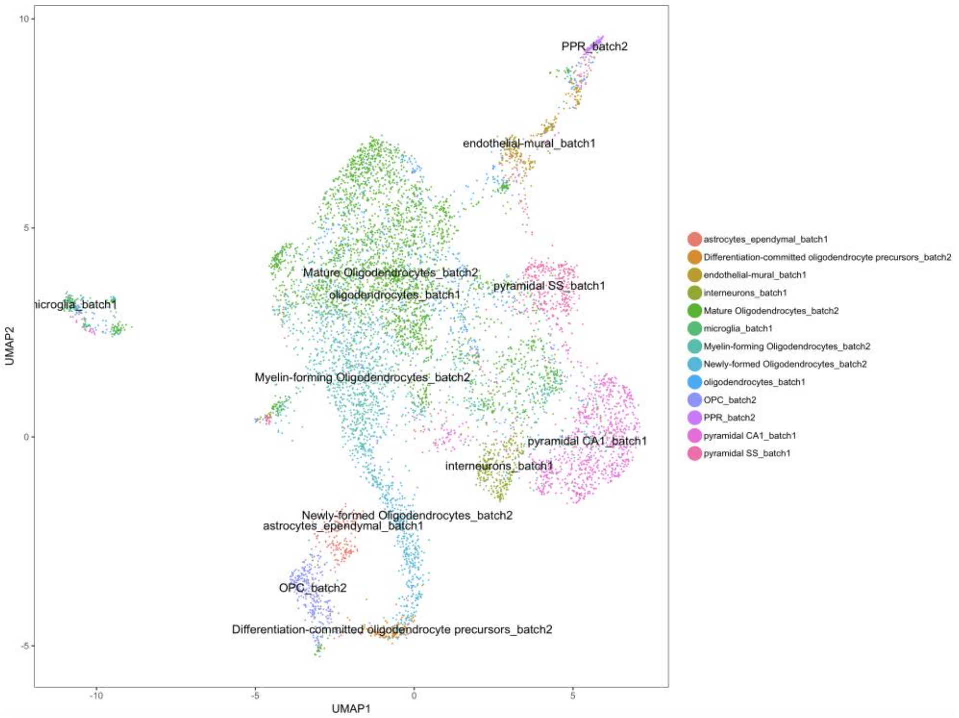

4.4 fastMNN

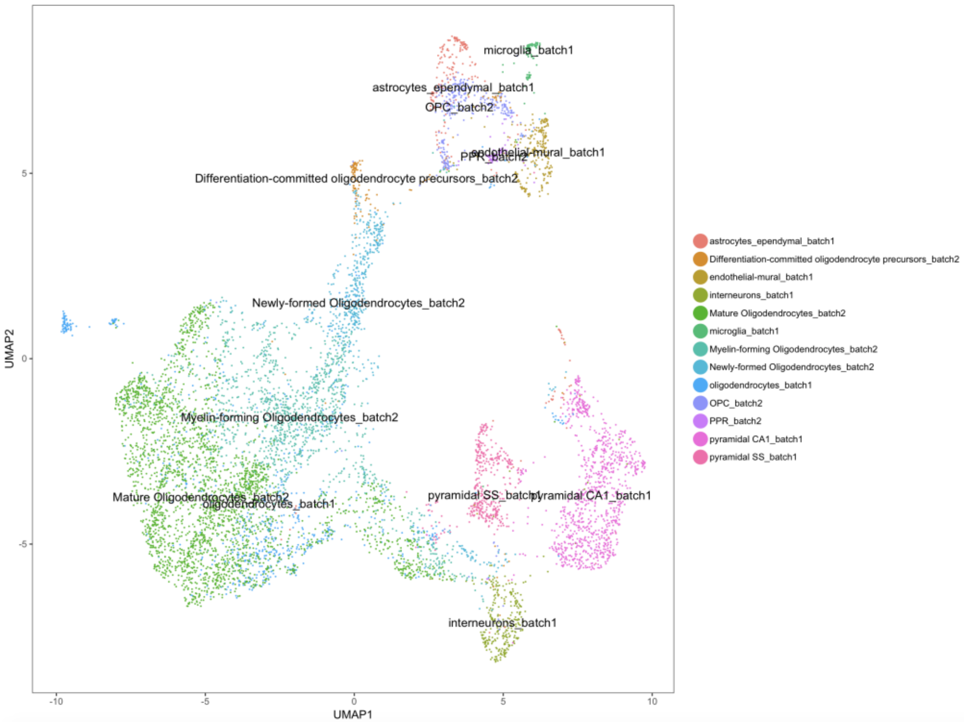

4.5 BBKNN

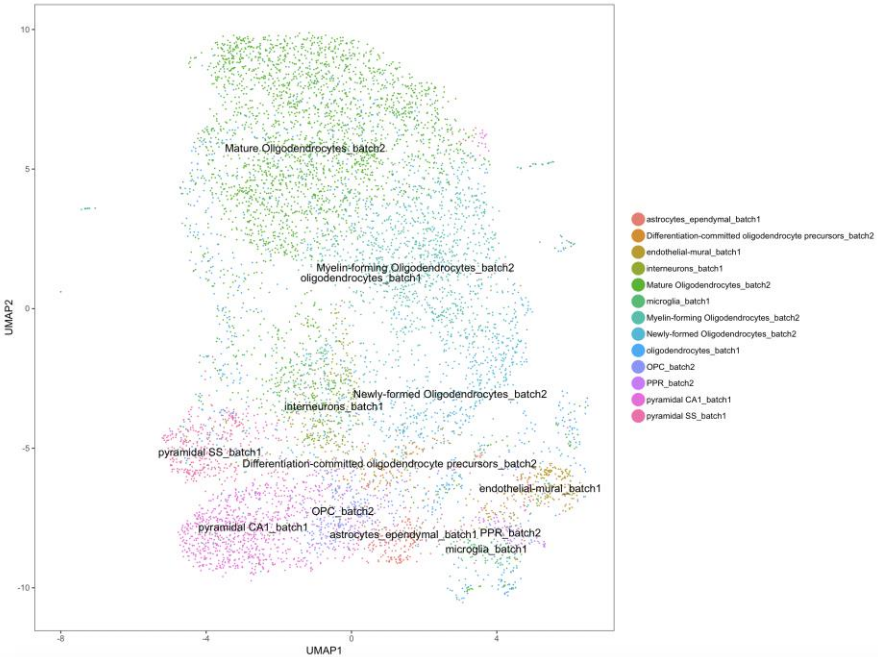

4.6 BEER (perplexity=5, cell number per group=10)

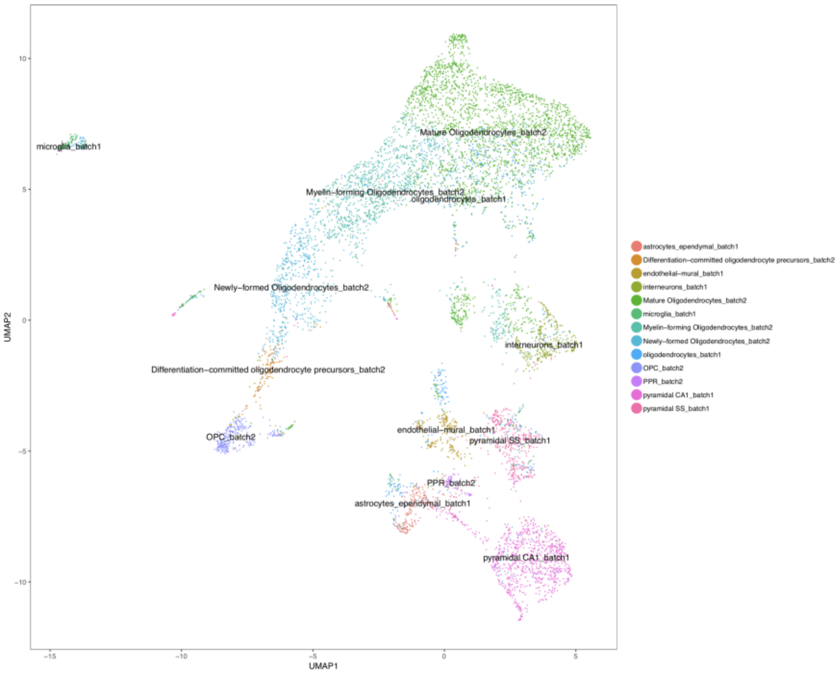

4.7 BEER (perplexity=50, cell number per group=10)

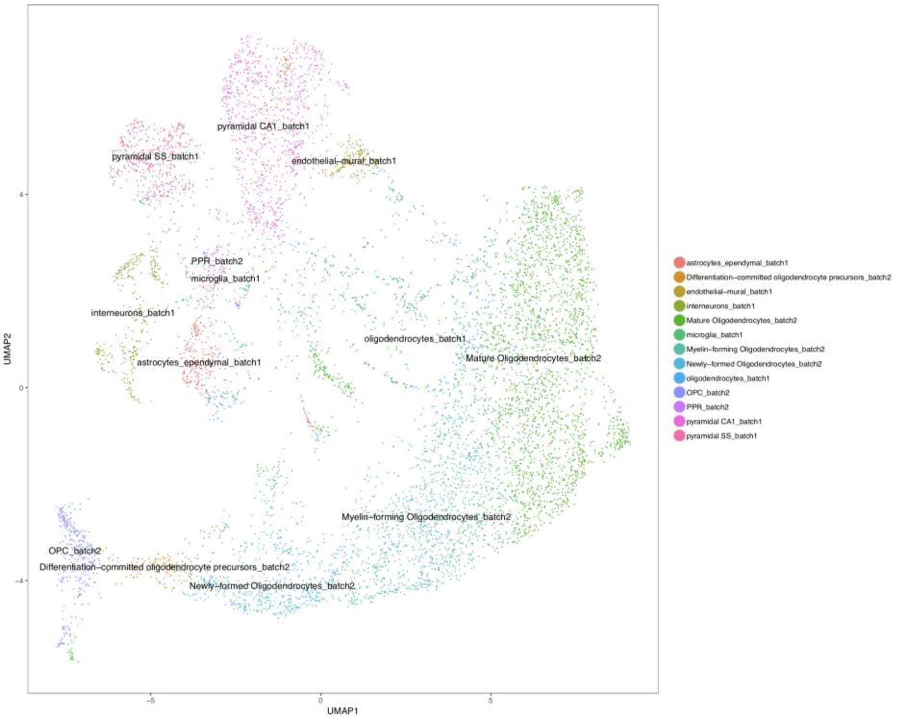

4.8 BEER (perplexity=100, cell number per group=10)

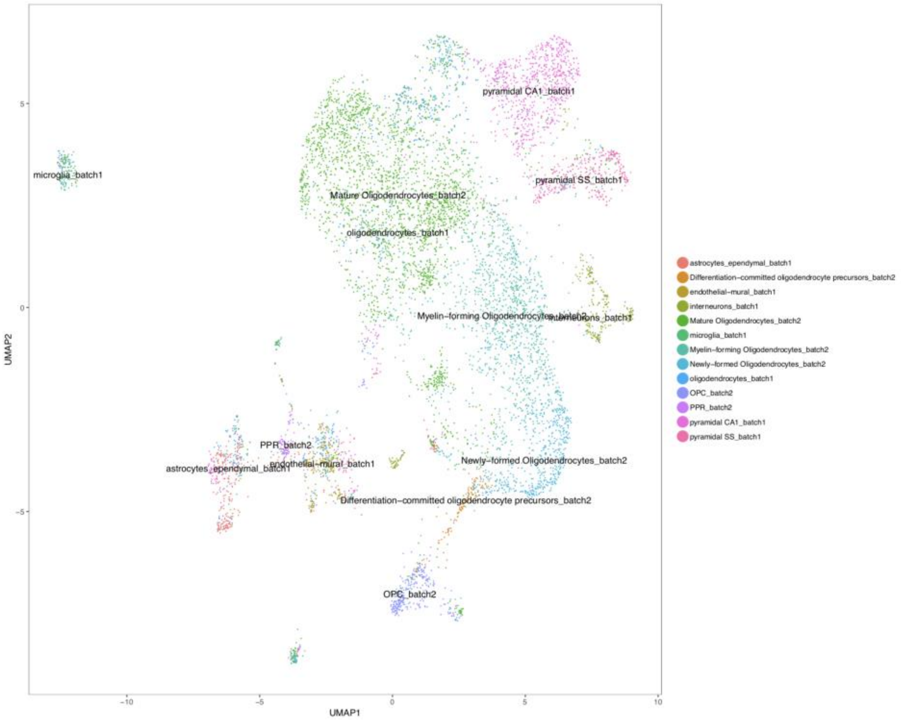

4.9 BEER (perplexity=30, cell number per group=50)

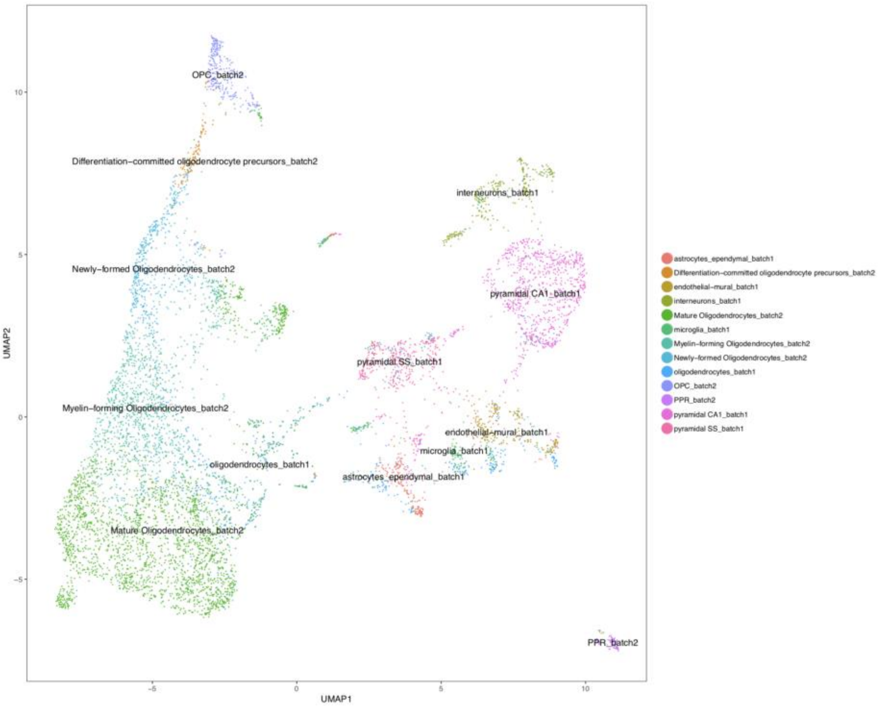

5.0 BEER (perplexity=30, cell number per group=100)

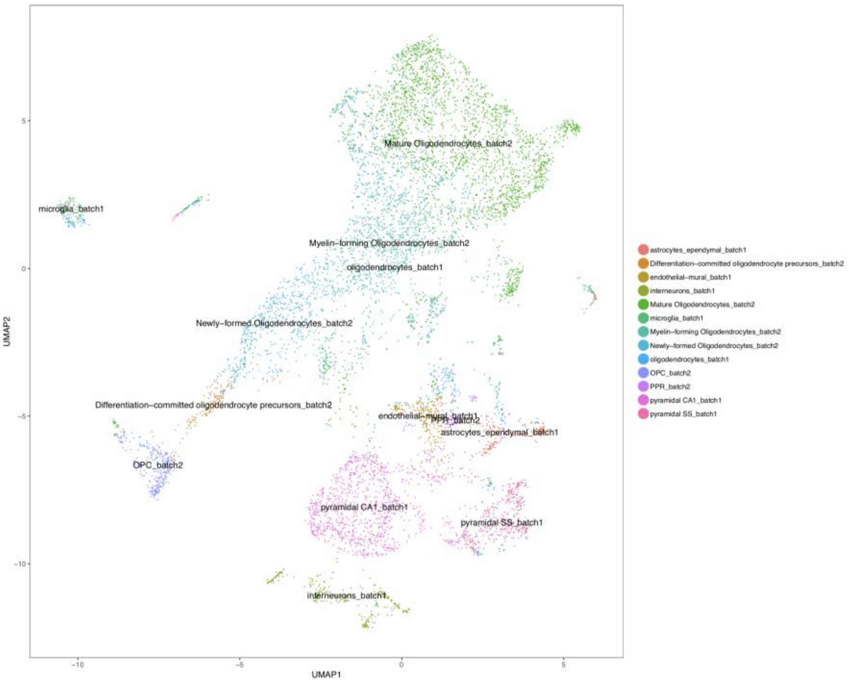

5. Silhouette Plot

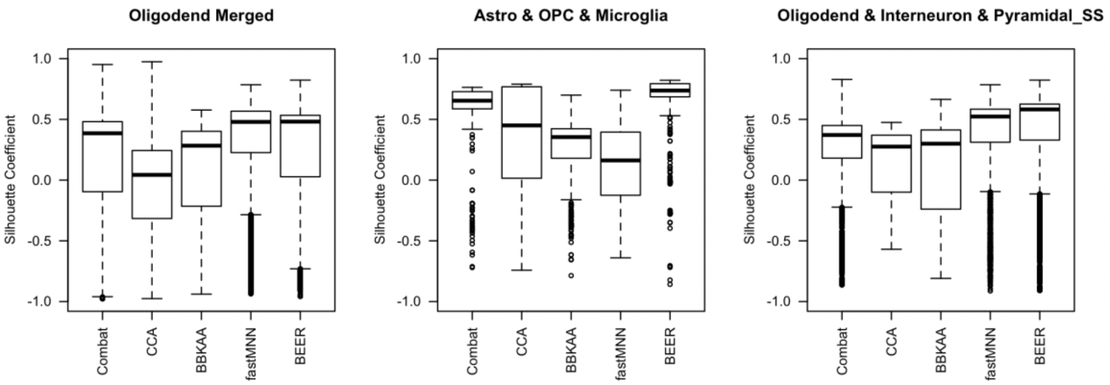

We use "silhouette" function in R to calculate Silhouette Coefficient, which can be used to evaluate the distance between different-type cells. Higher Silhouette Coefficients indicate different-type cells are better separated. In the above figure, "Oligodend Merged" means that we merge the oligodendrocytes of Batch1 and Batch2 into one cluster (oligodendrocyte is the only cell type shared by those two batches) and use all cell types of two batches to draw this plot. "Astro & OPC & Microglia" means that we only use astrocytes, OPC, and microglia to draw this plot. "Oligidend & Interneuron & Pyramida_SS" means that we use oligodendrocytes, interneuron, and pyramidal SS cells to draw this plot. In all these three benchmarks, BEER achieves high Silhouette Coefficients.

6. User Guide of BEER

GitHub: https://github.com/jumphone/BEER.

6.1 Requirement

Please install R (>=3.5), and install two packages: "Seurat" and "pcaPP"

Please install Python, and install one package: "umap-learn"

Install R: https://www.r-project.org/

Install Seurat (in R): install.packages('Seurat') # version 2.3.4

Install pcaPP (in R): install.packages('pcaPP') # version 1.9-73

Install umap-learn (python): pip install umap-learn

6.2 Usage

Step1. Load Data

#################### R script start ####################

library(Seurat)

```
source('https://raw.githubusercontent.com/jumphone/BEER/master/BEER.R')
#Load Demo Data (subset of GSE70630: MGH53 & MGH54)
#Download: https://github.com/jumphone/BEER/raw/master/DATA/demodata.zip
D1 <- read.table(unz("demodata.zip","DATA1_MAT.txt"), sep='\t', row.names=1, header=T)
D2 <- read.table(unz("demodata.zip","DATA2_MAT.txt"), sep='\t', row.names=1, header=T)
# "D1" & "D2" are UMI matrix (or FPKM, RPKM, TPM, PKM ...; Should not be gene-centric scaled data)
# Rownames of "D1" & "D2" are gene names
# Colnames of "D1" & "D2" are cell names
#################### R script end ####################
```

Step2. Detect Batch Effect

```
#################### R script start ####################
mybeer <- BEER(D1, D2, CNUM=10, PCNUM=50, CPU=2)
par(mfrow=c(1,2))
plot(mybeer$cor, xlab='PCs', ylab='PCC', pch=16)
plot(-log(mybeer$fdr,10), xlab='PCs', ylab='-log10(FDR)', pch=16)
#################### R script end ####################
```

Step3. Visualization

```
#################### R script start ####################
pbmc <- mybeer$seurat

# Keep batch-effect

ALLPC <- 1:length(mybeer$cor)
pbmc <- RunUMAP(object = pbmc, reduction.use='pca',dims.use = ALLPC,
check_duplicates=FALSE)
```

DimPlot(pbmc, reduction.use='umap', group.by='batch', pt.size=0.1)

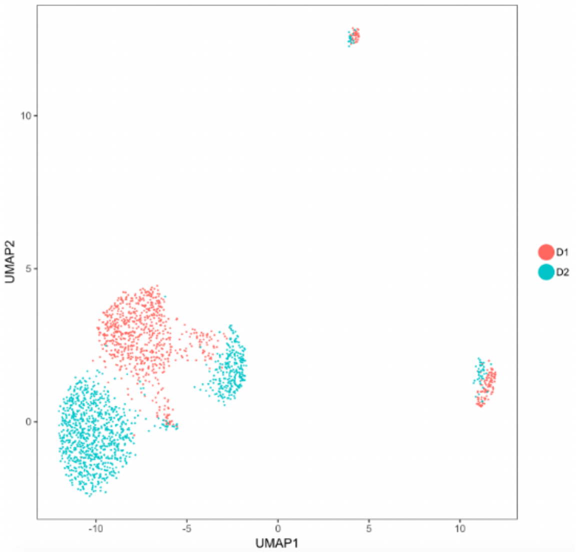

Remove batch-effect

PCUSE <- which(mybeer$cor> min(0.7, median(mybeer$cor))   & mybeer$fdr<0.05)

Users can set the cutoff of "mybeer$cor" based on the distribution of "mybeer$cor".

pbmc <- RunUMAP(object = pbmc, reduction.use='pca',dims.use = PCUSE,

check_duplicates=FALSE)

DimPlot(pbmc, reduction.use='umap', group.by='batch', pt.size=0.1)

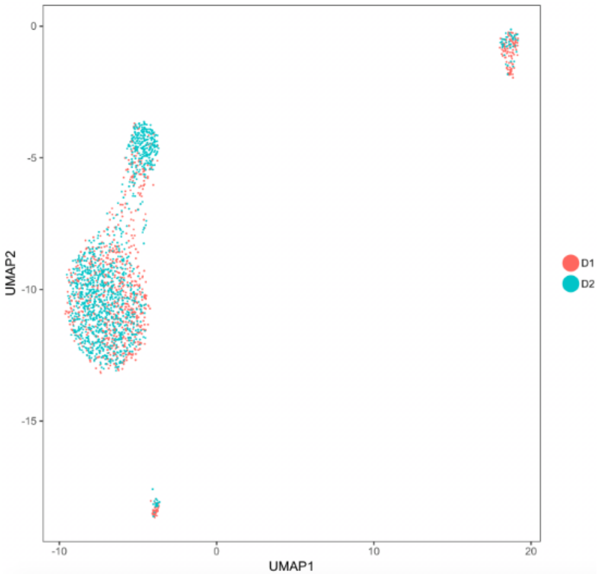

#################### R script end ####################

7. BEER with multiple batches

we provide a new function named MBEER to deal with multiple batches, https://github.com/jumphone/BEER#ii-combine-multiple-batches. MBEER implements the iteration of "Combine Two Batches". It compares each batch with the batch having the largest cell number, with the assumption that the batch having the largest cell number is likely to include largest number of cell-types within all batches. In MBEER, users can also define a batch as the batch with largest number of cell types by labeling that batch with "MAXBATCH". Then, all the other batches will be compared with the "MAXBATCH".

8. Detect PCs with both batch effects and biological variances

Users can use the result of BEER to check whether the removed PCs have biological meaning or not. If users find those removed PCs have biological meaning, then they can use other methods, such as ComBat, to modify those PCs.

Here is a demo (two oligodendroglima samples) of inspecting whether a PC removed by BEER has biological meaning.

After obtaining the BEER object (default name is "mybeer") by following the instruction of our website (https://github.com/jumphone/BEER), users can use the following command options to visualize the batch effect of each PC:

plot(mybeer$cor, xlab='PCs', ylab="COR", pch=16)

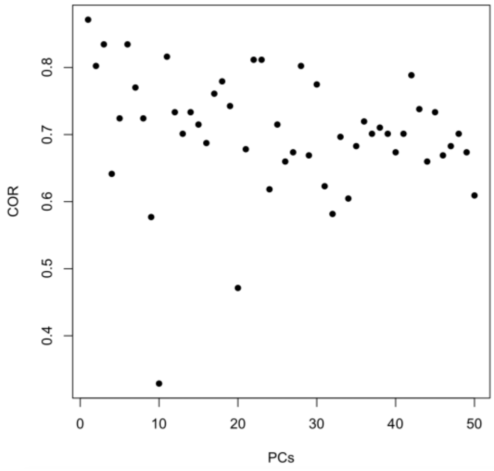

The above result shows that PC10 has the strongest "batch effect". Then, users can use the Seurat object of mybeer (2.3.4, not for Seurat 3.0) to extract PC10 related genes.

TOP=names(sort(mybeer$seurat@dr$pca@gene.loadings[,10], decreasing=TRUE)[1:100])

write.table(TOP, file='TOP100.txt', quote=F, row.names=F, col.names=F, sep='\t')

Finally, users can use some enrichment method to test the biological meaning of those genes. Here is the enrichment result (KEGG) of PC10's signature genes by using ClueGO:

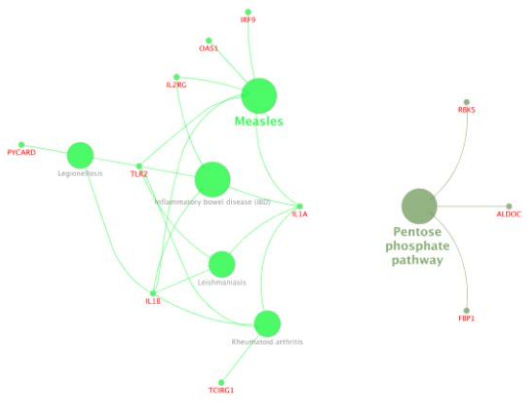

Those above pathways are related to PC10, and may be related to some biological variance that is co-occurring with batch effect.

References

Butler, A., *et al.* Integrating single-cell transcriptomic data across different conditions, technologies, and species. *Nat Biotechnol* 2018;36(5):411-420.

Haghverdi, L., *et al.* Batch effects in single-cell RNA-sequencing data are corrected by matching mutual nearest neighbors. *Nat Biotechnol* 2018;36(5):421-427.

Johnson, W.E., Li, C. and Rabinovic, A. Adjusting batch effects in microarray expression data using empirical Bayes methods. *Biostatistics* 2007;8(1):118-127.

Marques, S., *et al.* Oligodendrocyte heterogeneity in the mouse juvenile and adult central nervous system. *Science* 2016;352(6291):1326-1329.

Park, J.-E., *et al.* Fast Batch Alignment of Single Cell Transcriptomes Unifies Multiple Mouse Cell Atlases into an Integrated Landscape. *biorxiv* 2018.

Zeisel, A., *et al.* Brain structure. Cell types in the mouse cortex and hippocampus revealed by single-cell RNA-seq. *Science* 2015;347(6226):1138-1142.
